# Incidental diagnosis of Mazabraud syndrome presenting as a lower extremity soft tissue mass: A case report and literature review

**DOI:** 10.1016/j.ijscr.2024.110559

**Published:** 2024-11-04

**Authors:** Hannia Isabel Cortez Marquina, Joab Rafael Galvan Bustillos, Jean Enrique Pierzo Morales, Fernando Cordera González de Cosío

**Affiliations:** a American British Cowdray Medical Center, 154 Carlos Graef Fernandez Av, Central Tower, medical office 515, Mexico City 05300, Mexico; b Loma Linda University Health, 11175 Campus St, Loma Linda, California 92350, United States of America

**Keywords:** Mazabraud's syndrome, Intramuscular myxoma, Soft-tissue mass, Fibrous dysplasia, Case report

## Abstract

**Introduction and importance:**

Mazabraud's syndrome is a rare condition, describing the presence of fibrous bone dysplasia and intramuscular myxomas, with an incidence of 1:1,000,000. The aim of this article is to provide a review of the clinical presentation of Mazabraud's syndrome, including indications for surgical treatment, and follow-up strategies.

**Case presentation:**

A 46-year-old woman presented with a 3-month history of a painless mass in the right gluteal region, she referred a rapid increase in the mass's volume in the 3 weeks prior to consultation. Physical examination revealed a solid, non-mobile mass adhered to the deep tissues of the right gluteal region. Magnetic resonance imaging (MRI) identified a probable intramuscular myxoma in the right gluteus, and incidentally evidenced an area suggestive of fibrous dysplasia in the left iliac bone. A total resection of the soft-tissue was performed, obtaining clear surgical margins. Pathology study confirmed the diagnosis of intramuscular myxoma. More than one year after surgical resection, the patient remains asymptomatic and has satisfactory results.

**Clinical discussion:**

This case presented some unique features including a solitary myxoma, and contralateral fibrous dysplasia, surgical challenges included preoperative planning, as the myxoma was relatively large, and a careful resection to preserve the tumoral capsule, treatment included resection to attain clear margins, the sparing of healthy muscle proved to be valuable for a rapid postoperative recovery.

**Conclusion:**

The diagnosis of Mazabraud's syndrome can be overlooked given its very low incidence. Resection is warranted in symptomatic cases and because of the risk of late recurrence, long-term follow-up is required after surgery.

## Highlights

• Mazabraud syndrome is a rare clinical entity usually with an asymptomatic course.
• Surgical intervention may be warranted for patients with compressive symptoms generated by intramuscular myxomas.
• Most cases involve muscular and bone affection in the same extremity as a result of the mutation of the GNAS-1 gene.
• Long term follow-up with imaging is necessary given the potential for late intramuscular myxoma recurrence.

## 1 Introduction

Mazabraud's syndrome is defined as the simultaneous presentation of fibrous bone dysplasia and intramuscular myxomas [1]. It was first described by Henschen in 1926, and later refined by Mazabraud in 1967, who detailed the association between its two components [2,3]. The prevalence of this pathology is estimated to be 1:1,000,000. It affects women more frequently, and it typically presents during the fifth decade of life [1]. The most accepted pathophysiological model proposes that both the fibrous bone dysplasia and intramuscular myxomas develop as a result of a mutation of the GNAS-1 gene, also associated with McCune Albright syndrome and isolated fibrous bone dysplasia [4].

Clinically, the syndrome can present with soft tissue masses caused by multiple intramuscular myxomas, and pain or bone deformity and recurrent fractures as a consequence of fibrous bone dysplasia, with the lower extremities being most frequently affected [1,5,6].

This case has been reported in line with the SCARE 2023 criteria [7].

## 2 Case presentation

We present the case of a 46-year-old woman who sought medical evaluation in March of 2023 for the presence of a painless mass in the right gluteal region, referring concern about a recent rapid growth and discomfort. Upon questioning, the patient attributed the mass to several falls she experienced while exercising. She denied any past medical history and referred no other symptoms including lymphadenopathy or weight loss.

On physical examination, asymmetry and an increased volume were observed in the right gluteal region. On palpation, a hard and fixed tumor with well-defined edges was identified. The pulses, range of mobility, as well as motor and sensitive function were normal in the affected extremity, no lymphadenopathy was noted. Complete physical examination of the skin revealed no abnormalities.

**Fig. 1:**
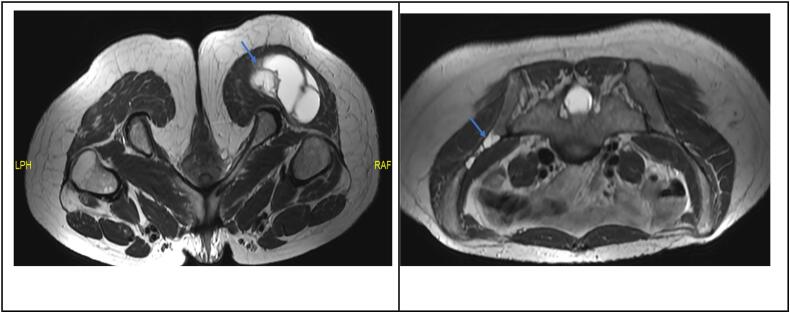
Magnetic resonance images (axial) pointing with blue arrows a well-defined lesion in the right gluteus maximus with a volume of 343 ml, and a left iliac bone lesion consistent with fibrous dysplasia. (For interpretation of the references to colour in this figure legend, the reader is referred to the web version of this article.)

The patient had an ultrasound of the gluteal region done, revealing a 12-cm tumor, with findings suggestive of a probable myxoma. Subsequently, a simple and contrast-enhanced magnetic resonance imaging (MRI) of the pelvis demonstrated a tubular lesion in the right gluteus maximus, with a volume of 343 cc, containing an extensive multiloculated cystic component and a nodular solid component in the superomedial area, findings also compatible with an intramuscular myxoma (Fig. 1). Additionally, a multilobulated cystic lesion was found in the left iliac bone with a longitudinal axis of 7.6 cm, consistent with fibrous dysplasia.

The case was discussed with a multidisciplinary committee, and a complete surgical resection of the tumor in the right gluteal region with intraoperative pathology and wound closure by reconstructive surgery was recommended.

**Fig. 2:**
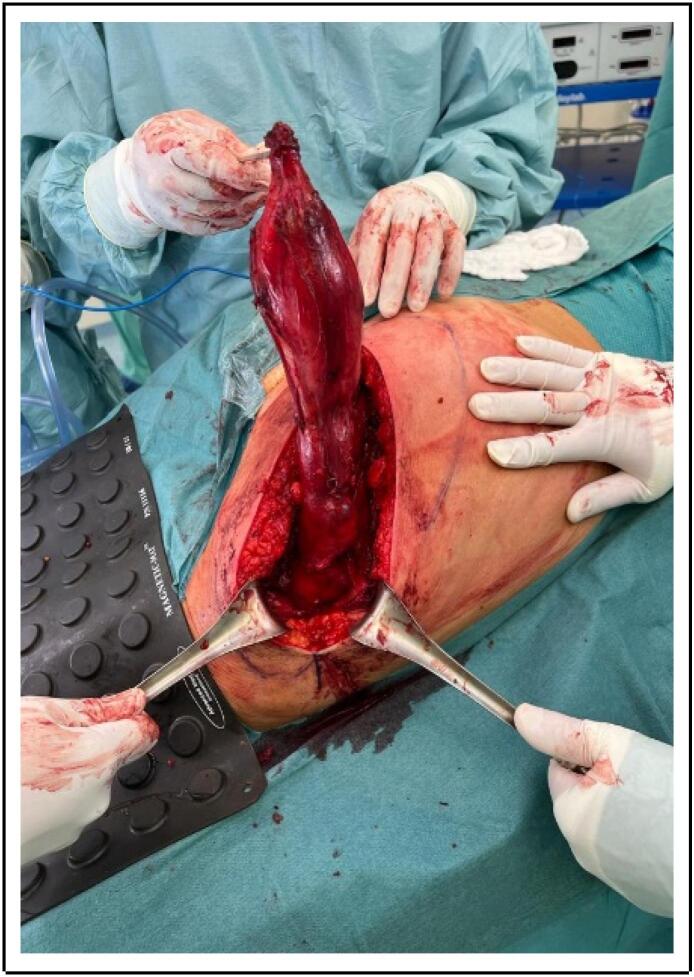
Surgical exposure of the tumor in the right gluteal region, prior to excision, note the intact capsule.

**Fig. 3:**
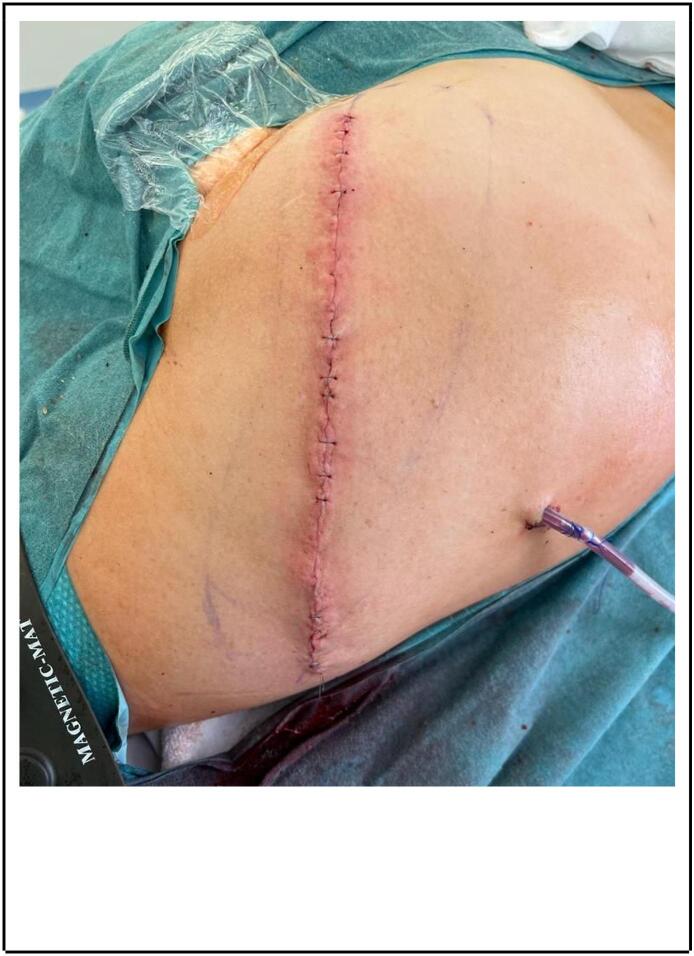
Final closure of the defect, no grafts or flaps were required.

Before the first incision, an ultrasound was made, and the tumor was properly delimited with a skin marker over an approximately 30-cm area. An oblique incision was performed with scalpel, followed by monopolar cautery, the gluteus maximus fascia was accessed in the direction of its fibers, exposing the tumor. A careful and meticulous resection was performed, separating the tumor from the surrounding muscle, taking special care of not disrupting the tumoral capsule (Fig. 2). During the resection the tumor was found close to, but not in contact with the sciatic nerve nor any major vascular structures, and it was completely resected in a single piece with an intact capsule, the specimen was marked for intraoperative pathological study, which reported a hypocellular tumor with extensive myxoid changes, and clear surgical margins confirming a completely resected myxoma. After resection, the surgical wound was copiously irrigated, assuring hemostasis, a Blake drain was positioned over the wound bed, and brought out through a separate skin incision. The gluteus maximus fascia was closed with continuous Vicryl 3–0 stitches, and subcutaneous tissue was closed with interrupted Monocryl 3–0 stitches, plastic surgery completed skin closure using subcuticular Monocryl 4–0 stitches, no skin flaps or grafts were required (Fig. 3).

**Fig. 4:**
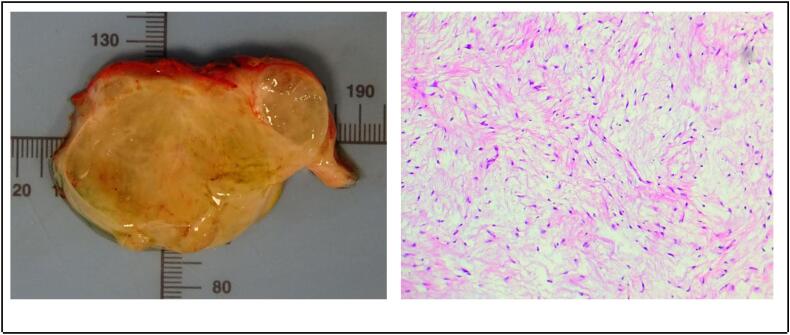
Specimen: 24 × 6 × 5 cm weighting 391.8 g. Histological analysis: Intramuscular myxoma, cellular variant, CD34 positive, S100 negative cells.

The patient had an excellent postoperative recovery, pain was adequately controlled with acetaminophen and ketorolac, achieving an early ambulation. After proper wound hygiene and drain training were discussed with the patient, she was successfully discharged on postoperative day 1, the drain was removed during a follow-up visit one week after surgery, during that visit, results from final pathology report were shared with the patient. Final pathology with immunohistochemistry showed diffuse CD34 positive in neoplastic cells, S100 negative, findings consistent with intramuscular myxoma (Fig. 4), the patient declined genetic testing. One year after the operation, the patient has an excellent aesthetic and functional result. Further plan for management and follow-up were discussed with the her and she will continue with oncological and orthopedic surgery follow-up with X-rays of the identified bony lesion every 6 months.

## 3 Discussion

Intramuscular myxomas are rare tumors by themselves, with an estimated incidence of 1:1,000,000 [8], and fibrous bone dysplasia has a higher incidence, estimated to be between 1:5000 and 1:10,000 [9], representing up to 7 % of benign bone tumors [10,11].

### 3.1 Epidemiology

The estimated prevalence of this syndrome in the general population is 1:1,000,000 with around 100 cases currently published in the literature, approximately 68 % of these cases correspond to women and 32 % to men [6]. It is generally diagnosed on the fifth decade of life, fibrous dysplasia typically precedes the development of intramuscular myxomas, with a mean age at diagnosis of 40 and 47 years respectively [1]. These lesions occur in the lower extremities in 83 % of cases, with involvement of both upper and lower extremities in 41 % of cases, the least frequently affected locations are the skull and thorax [6]. It is estimated that the prevalence of this syndrome in patients with an initial diagnosis of fibrous dysplasia is between 1 and 2.4 % [2].

### 3.2 Clinical presentation

Patients with Mazabraud's syndrome may remain asymptomatic until adulthood. Typical symptoms include a clinically apparent soft-tissue tumor, pain, or compression of adjacent structures by intramuscular myxomas, and atypical fractures secondary to fibrous dysplasia [12].

Intramuscular myxomas most frequently develop as slow-growing, asymptomatic, multiple soft-tissue tumors located in large muscle groups, the most commonly affected muscle is the quadriceps [1,13]. Fibrous dysplasia usually involves multiple bones, with the femur and pelvis being the most affected [1].

A multicenter study by Majoor et al. [2] recorded one of the largest series of patients with Mazabraud's syndrome, including thirty-two patients from 6 centers diagnosed between 1980 and 2015. The prevalence of Mazabraud's syndrome was 2.2 % in the combined cohort of 1446 patients with fibrous dysplasia. The results confirmed that in most cases fibrous dysplasia affects multiple bones (84 %), the most frequent location of myxomas included the quadriceps (65.6 %), adductors (34.4 %) and glutes (21.9 %) and in the 97 % of patients, myxomas were found adjacent to the area affected by fibrous dysplasia. The most common symptoms included pain (66 %) and painless intramuscular tumor (16 %). Our patient referred only discomfort, rather than pain generated by the intragluteal mass, the fibrous bone dysplasia was an incidental finding, she had no previous history of pain, bone deformity or fractures.

### 3.3 Pathophysiology

Mazabraud's syndrome is classified within the spectrum of alterations associated with fibrous dysplasia, originated by a mutation in the GNAS-1 gene. Genetic analysis in patients with this pathology has shown an activating mutation in codon 11 of the GNAS-1 gene, with differential cellular involvement and varying degrees of mosaicism. This gene encodes the alpha subunit of the Gs protein, responsible for regulating levels of cyclic adenosine monophosphate (cAMP) participating in intracellular signaling. Similar mutations have been found in cases of isolated fibrous dysplasia and fibrous dysplasia in association with endocrinological alterations including McCune Albright syndrome [14].

Activating mutations of the GNAS-1 gene result in excessive production of cAMP, which causes abnormal bone synthesis in osteoprogenitor cells, with a fibrous stroma structurally inadequate and prone to fractures [12]. Both characteristic lesions of Mazabraud's syndrome have been found to carry a mutation in the GNAS-1 gene [3].

The currently available evidence suggests that the association with intramuscular myxomas is more frequent in cases of polyostotic fibrous dysplasia, possibly due to a more generalized tissue distribution of the mutation in the GNAS-1 gene, extending to soft tissues [2].

### 3.4 Diagnosis

Magnetic resonance imaging (MRI) is generally used to identify intramuscular myxomas. MRI features include tumors with well-defined borders, showing a low intensity signal on T1 and high intensity on T2. With the administration of contrast agent, these tissues usually are poor vascularized [15]^.^

Imaging studies are also useful for identifying areas of fibrous bone dysplasia, including complete skeletal evaluation with x-rays, which may reveal ground-glass-appearing radiolucent lesions surrounded by condensed bone. The edges of the lesion are usually well defined and the cortex is intact, however it may be thinned due to the expansive nature of the lesion [14,15]. In our case, since the fibrous bone dysplasia was identified and characterized with an MRI, we did not add an x-ray to the initial evaluation.

The use of fluorodeoxyglucose positron emission tomography (FDG PET-CT) has been described for identifying lesions associated with Mazabraud's syndrome. The maximum Standardized Uptake Value (SUVmax) for intramuscular myxomas ranges from 1.3 to 2.6, while in areas of fibrous dysplasia, it varies from 3.3 to 19.2 [15]. Given the absence of systemic symptoms and the very low likelihood of disseminated disease in our patient, we did not consider a FDG PET-CT was warranted.

Definitive diagnosis requires histopathological confirmation from a sample obtained either through a needle biopsy or after the excision of intramuscular myxomas. The histological analysis usually reveals hypocellular lesions with a myxoid matrix, loose reticular fibers with scattered collagenous and vascular structures, and spindle-shaped cells with hyperchromatic, pyknotic nuclei and scant cytoplasm. On pathological analysis, areas of fibrous bone dysplasia are characterized by the replacement of normal bone tissue with fibrous tissue and irregular spicules of immature bone [15].

Differential diagnoses include benign lesions such as myxolipomas, myxoid neurofibromas, and myxochondromas, as well as some malignant lesions, including myxoid liposarcomas, malignant myxoid histiocytomas, and low-grade fibromyxoid sarcomas [14].

### 3.5 Treatment

The approach for asymptomatic cases is limited to clinical and radiological follow-up [5]. Medical treatment with non-steroidal anti-inflammatory drugs is indicated for pain associated with fibrous bone dysplasia. In the cases of moderate, severe, and persistent pain, the use of bisphosphonates has been proposed, although there is no evidence of any benefit in preventing lesion progression [16]. After discussing with the patient, she opted not to take medical treatment given the absence of symptoms.

Surgical resection of intramuscular myxomas is warranted in cases presenting with pain or compressive symptoms [11]. Currently, there is no consensus regarding the optimal surgical margins due to evidence of microscopic infiltration into adjacent muscle tissue, despite the well-defined margins typical of these tumors. Most authors recommend simple excision with a margin that includes some muscle fibers, as larger resections may result in greater morbidity [17]. We opted for a simple excision including the tumoral capsule, and some adjacent muscle fibers, this resulted in a rapid recovery after surgery.

Surgical treatment of fibrous dysplasia may be justified for correcting bone deformities, preventing pathological fractures, or addressing painful lesions. The need for surgical intervention is more frequent for lesions located in the lower extremities, and procedures such as intra-lesional excision of the tumor in the bone followed by cryosurgery, and correctional osteotomy followed by homologous bone transplantation and fixation have been described [11].

### 3.6 Prognosis

Local and regional recurrence of intramuscular myxomas has been documented [18]. Recurrence usually presents on average 10 years after the initial resection and is more frequent in cases of tumors with a greater cellularity [17]. For this reason, it is recommended to continue long-term follow-up [18]. In currently available reports, recurrent lesions are benign, with no evidence of a potential for malignant transformation [19].

There is an increased risk for the development of osteosarcoma in bones affected by fibrous dysplasia, reaching 8.3 % in patients with Mazabraud's syndrome in contrast with a less than 1 % risk in patients with isolated fibrous dysplasia [19]. Follow-up with x-rays at 6-month intervals is recommended, an initial FDG PET-CT has been recommended by some authors given the potential for malignant transformation, however, it is not routinely warranted [18].

## 4 Conclusion

Mazabraud's syndrome has an asymptomatic course in most cases; treatment options must be individualized according to each patient's characterystics, ranging from symptomatic management to surgical treatment. Long-term follow-up is required both after surgical resection and for surveillance-only cases, due to the risk of recurrence for intramuscular myxomas and the risk of sarcoma development in bones affected by fibrous dysplasia. Some limitations of this case include the patient's decision to decline a genetic study, and the relatively short follow-up period after surgery to date. Strengths included an adequate multidisciplinary clinical decision-making and management, underscoring the importance of an adequate knowledge of the different aspects of Mazabraud's syndrome.

## Research registration number

1.Name of the registry: Research Registry

2.Unique identifying number or registration ID: researchregistry10727

3.Hyperlink to your specific registration (must be publicly accessible and will be checked): https://www.researchregistry.com/browse-the-registry#home/

## Guarantor

FC

## CRediT authorship contribution statement

HC (Corresponding author): data collection, study concept, writing the paper, data interpretation.

JG: study concept, data collection, data interpretation, writing the paper.

JP: data collection, writing the paper.

FC: data interpretation, study concept, writing the paper.

## Consent

Written informed consent was obtained from the patient for publication of this case report and accompanying images.

## Ethical approval

Ethical approval for this work was given by the “Research Ethics Committee” at our institution.

## Sources of funding

This research did not receive any specific grant from funding agencies in the public, commercial, or not-for-profit sectors.

## Declaration of competing interest

All authors declare no conflicts of interest.
